# Neural Correlates of Erotic Stimulation under Different Levels of Female Sexual Hormones

**DOI:** 10.1371/journal.pone.0054447

**Published:** 2013-02-13

**Authors:** Birgit Abler, Daniela Kumpfmüller, Georg Grön, Martin Walter, Julia Stingl, Angela Seeringer

**Affiliations:** 1 Department of Psychiatry and Psychotherapy, University of Ulm, Ulm, Germany; 2 Department of Psychiatry, Otto von Guericke-University, Magdeburg, Germany; 3 Institute of Pharmacology of Natural Products & Clinical Pharmacology, University of Ulm, Ulm, Germany; University of Cambridge, United Kingdom

## Abstract

Previous studies have demonstrated variable influences of sexual hormonal states on female brain activation and the necessity to control for these in neuroimaging studies. However, systematic investigations of these influences, particularly those of hormonal contraceptives as compared to the physiological menstrual cycle are scarce. In the present study, we investigated the hormonal modulation of neural correlates of erotic processing in a group of females under hormonal contraceptives (C group; N = 12), and a different group of females (nC group; N = 12) not taking contraceptives during their mid-follicular and mid-luteal phases of the cycle. We used functional magnetic resonance imaging to measure hemodynamic responses as an estimate of brain activation during three different experimental conditions of visual erotic stimulation: dynamic videos, static erotic pictures, and expectation of erotic pictures. Plasma estrogen and progesterone levels were assessed in all subjects. No strong hormonally modulating effect was detected upon more direct and explicit stimulation (viewing of videos or pictures) with significant activations in cortical and subcortical brain regions previously linked to erotic stimulation consistent across hormonal levels and stimulation type. Upon less direct and less explicit stimulation (expectation), activation patterns varied between the different hormonal conditions with various, predominantly frontal brain regions showing significant within- or between-group differences. Activation in the precentral gyrus during the follicular phase in the nC group was found elevated compared to the C group and positively correlated with estrogen levels. From the results we conclude that effects of hormonal influences on brain activation during erotic stimulation are weak if stimulation is direct and explicit but that female sexual hormones may modulate more subtle aspects of sexual arousal and behaviour as involved in sexual expectation. Results may provide a basis for future imaging studies on sexual processing in females, especially in the context of less explicit erotic stimulation.

## Introduction

Sexual function and dysfunction are modulated by various physical, psychological, social, and also pharmacological conditions. Among these, it has been shown that changing hormonal levels in females, either physiologically during the menstrual cycle, after the menopause, or induced by oral contraceptives have a great impact [1]. The female hormonal cycle is characterized by rising estrogen levels in the follicular phase from basal levels during menstruation to their peak at mid-cycle ovulation. Ovulation related to peaking luteinizing hormone levels promotes the rise of progesterone levels that remain high during the luteal phase and fall steeply towards the end of the cycle. Accordingly, cyclicity of sexual interest has been shown in several studies with rising sexual interest during the follicular phase while estrogen levels increase, a peak around ovulation and a decline during the luteal phase, most pronounced towards its end [2], [3] (review in [1], but see also [4]).

Taking hormonal contraceptives into account, the multidimensionality of interacting psychological and physiological effects on women’s sexual behaviour and experiences becomes even more evident [5]. Psychologically, when compared against less safe contraceptive methods, intake of hormonal contraceptives appears to be associated with a decrease in the anxiety of getting pregnant, thereby separating procreational from recreational sexuality and increasing women’s enjoyment of sex [1] as well as their frequency of sexual activity [6]. Physiologically, however, it has also been reported in the past, that hormonal contraception may reduce sexual drive in women [7] although more recent reports are controversial [8]. In particular, modification of androgen-levels [9] and loss of estrogen fluctuation have been suggested to decrease sexual desire and vaginal lubrication [5].

In recent years, the increasing recognition of female sexuality and also the desire to tackle hormonally, pharmacologically or otherwise induced sexual dysfunction has promoted investigations beyond studies of behaviour and laboratory measures on neurobiological correlates of female sexual functioning. Investigations using functional imaging first postulated decreased cerebral activation in women compared to men upon visual erotic stimulation [10], [11]. However, subsequent investigations demonstrated that brain activation particularly in the cingulate cortex, insula and orbitofrontal cortex increases with increasing estrogen levels during the follicular phase. In these networks, previously identified as relevant for sexual processing in animal studies [12] and male subjects [13], women around ovulation showed similar brain activation upon erotic stimulation as men [14]. Also, a combined estrogen and androgen therapy in ovariectomized women increased cerebral responsiveness for erotic stimuli, particularly in limbic regions [15]. However, a recent study in young women with regular cycles demonstrated decreased activation of both cortical and subcortical brain regions around the time of ovulation when compared against functional imaging data obtained during menses or at a time-point far apart from ovulation or menstruation [16]. Furthermore, hormonal influences on brain activation were not only demonstrated upon erotic stimulation but also for the processing of emotional pictures [17] or monetary rewards [18].

Very recently, we have investigated effects of different antidepressant drug classes on sexual functioning and cerebral activation during processing of erotic stimuli in men [19]. Since antidepressants are even more frequently prescribed to females [20] with similar but also distinct side effects on sexual functioning as observed in men, it appeared worthwhile to study these antidepressive medication effects also in women.

However, in females strict control of hormonal influences appears mandatory in order to rule out potential moderator effects. Particularly, effects of hormonal contraceptives on brain activation upon erotic stimulation have not yet been investigated although appearing ideal to obtain hormonal steady states. Therefore, it was the goal of this study to investigate the effects of hormonal contraceptives on brain activation during various conditions of erotic stimulation, and how these effects would compare against results obtained from a different group of women not taking hormonal contraceptives and who were measured during their mid-follicular and mid-luteal phase. Since brain activation upon erotic stimulation may vary with its form and intensity (e.g. explicit sexual intercourse or mere expectation of erotic stimuli) we implemented three different conditions of erotic stimulation, all of which have already been demonstrated to reliably elicit fMRI hemodynamic responses as an estimate of cerebral activation in brain areas associated with sexual processing [19], [21]. In the two conditions with rather explicit and direct stimulation, erotic video clips or pictures were presented. From the findings of previous studies [13] we inferred that these functional challenges would drive brain activation in a set of areas including the hypothalamus, thalamus, amygdala, anterior cingulate gyrus, insula, fusiform gyrus and precentral gyrus. For testing effects of less direct stimulation, subjects’ brain activation upon the anticipation of erotic picture stimuli was measured to investigate hormonal influences on the appetitive aspects of sexual processing beyond mere perception and associated processing. Again, expecting erotic stimuli has already been shown to elicit activation in brain regions related to the processing of rewards and mediating arousal [22]. Although different in the specific task characteristics, a previous study on classical conditioning using erotic pictures observed involvement of the anterior cingulate cortex, insula, amygdala and the ventral striatum responding on the conditioned stimulus with activation modulated by gender and awareness of contingency [23]. We therefore expected effects in similar networks.

## Materials and Methods

### 1. Subjects

24 healthy female, heterosexual subjects between 20 and 29 years of age (mean 24.0, SD 2.0) with a regular menstrual cycle were included in the study and completed the experiment. 12 of them regularly had taken oral contraceptives (C group) for the past 6 months. The other 12 women had not been under hormonal treatment for at least 6 months (nC group). All subjects were students at Ulm University or the University for Applied Sciences in Ulm. Groups did not differ regarding age (t(12) = 0.90, p = 0.38). None of the subjects smoked, one subject in each group was left-handed. The mean body mass index in the nC group was 24.9 (SD 4.3) and 20.8 (SD 1.0) in the C group. Four subjects in the C group took second or third generation contraceptives containing a combination of 0.03 mg ethylestradiol and 0.13 mg levonorgestrel or 0.15 mg desogestrel. The remaining 8 women took fourth generation contraceptives containing 0.03 mg ethylestradiol combined with 2 mg chlormadinone, 3 mg drospirenone, or 2 mg dienogest. C group subjects took the hormonally active contraceptive for up to 63 consecutive days and were instructed not to take the placebo pill included in some commercially available oral contraceptive regimen. This was done to reach steady state hormonal conditions before scanning.

All subjects in the nC group were scanned twice, once during the follicular phase at day 3 to 6 after onset of the menstruation (nC-F) and once during the luteal phase at day 6 to 10 after ovulation (nC-L). Subjects were handed commercially available luteinizing hormone test strips to assess ovulation. Phases of the menstrual cycle at the time of scanning were confirmed by blood samples of estradiol and progesterone levels analyzed in a laboratory using an electrochemiluminescence immunoassay (ECLIA) obtained from Roche Diagnostics (Mannheim; Germany). A priori, 6 subjects had been randomly assigned to be scanned first during the follicular phase, and then during the luteal phase. For the other six subjects the reversed schedule was applied. To account for MR imaging twice with possible habituation effects due to the longitudinal design in the nC group, MR imaging in the C group was also conducted a second time in half of the subjects. Thus, all the C group subjects were measured once after day 30 of taking oral contraceptives. Six subjects of this group were randomly selected and were scanned again after at least 14 days. From these subjects, only data from their second scan were included, complementing the first and only scan data from the other six subjects of the C group. Data from these twelve subjects was the entire final data set for the ensuing analysis.

The research has been approved by the ethics committee of Ulm University (Universität Ulm, Ethikkommission, 89069 Ulm). All participants gave written informed consent after full explanation of the study procedures and according to the principles expressed in the Declaration of Helsinki. Exclusion criteria were any psychiatric, neurological and major medical diagnoses currently or in the past, use of illegal drugs, excessive consumption of caffeine or alcohol as well as sexual dysfunction. Upon enrolment, subjects were asked for any problems concerning sexual arousal, libido and ability to achieve orgasm. They were asked to explain whether the stimulus material to be used (grown-up heterosexual couples practicing sexual intercourse) was in accord with their primary sexual preference. Upon each experimental day subjects completed a German version [24] of the Massachusetts General Hospital Sexual Function Questionnaire, MGH-SFQ [25], a self-rating questionnaire for the assessment of subjective sexual dysfunction. Standard MGH-SFQ questions asking about interest in sex compared to normal over the past month were slightly adapted, replacing the anchor “over the past month” with the anchor “over the past week” to match study conditions. Ratings on the SFQ range from 1 (improved sexual functioning) over 2 (normal sexual functioning) to 6 (absent sexual functioning). Subjects also completed a self-report scale on depressive symptoms, the Center for Epidemiologic Studies Depression Scale, CES-D [26], in its German language version, ADS (Allgemeine Depressions-Skala, [27].

### 2. Task and Stimuli

Two separate sessions were performed on one experimental day. In one session, erotic video clips as in a previous study [19] were presented alternating with neutral video clips in a standard block design with prolonged visual stimulation (see supplementary Figure S1). Erotic video clips demonstrated sexual interactions between one man and one or two women (petting, oral sex and vaginal intercourse) extracted from commercial adult films. The neutral video clips depicted men and women in emotionally neutral, non-erotic interactions (at a shop, at the airport). Nine different video clips of each type were presented at a length of 20 sec each, separated by a 20 sec interstimulus interval with a white fixation cross on a black screen. Video clips were presented in a pseudo-randomized order with no more than two consecutive clips of the same type (erotic/non-erotic).

The other session used another established paradigm [21], that has been demonstrated to reliably induce sexual arousal and to elicit hemodynamic responses in brain areas relevant for sexual and emotional arousal. Stimuli comprised 20 erotic and 20 non-erotic pictures of positive emotional content taken from the international affective picture system (IAPS, see supplementary Figure S2)) [28]. Erotic pictures depicted one man and one woman in erotic poses, control pictures showed people engaging in positively, emotionally laden, but non-erotic activities. Pictures were matched for standard values of arousal, pleasantness and dominance as provided from the IAPS and for sexual intensity as described before [21]. Stimuli were presented for 4 sec followed by a variable interstimulus interval with presentation of a white fixation cross for 7.5–10.5 sec. Ten stimuli of each type (erotic, non-erotic), were announced by the presentation of an arrow at a duration of 3–5 sec. Downward arrows predicted erotic, upward arrows predicted non-erotic stimuli. This aspect of the second session represents the third (expectation) condition studied. The paradigms used in the two sessions are depicted in the supplementary Figures S1 and S2. After completion of the second scanning session, subjects were asked outside of the scanner to rate each of the erotic and non-erotic picture stimuli for their impact on inducing subjective sexual arousal, measured by a numeric rating scale ranging from 1 to 9 (1: not sexually arousing at all, 9: very sexually arousing).

### 3. fMRI Acquisition

A 3.0 Tesla Magnetom ALLEGRA Scanner (Siemens, Erlangen, Germany) equipped with a head coil was used to acquire T1 anatomical volume images (1×1×1 mm voxels) and functional magnetic resonance images. 23 transversal slices were acquired with an image size of 64×64 pixels and a field of view of 192 mm. Slice thickness was 3 mm with 0.75 mm gap resulting in a voxel size of 3×3×3.75 mm. Images were centered on basal structures of the brain including subcortical regions of interest (basal ganglia and prefrontal regions). Functional images were recorded using a T2*-sensitive gradient echo sequence measuring changes in BOLD-contrast. In two different sessions, 487 and 552 volumes were obtained during viewing of video clips and IAPS pictures respectively, at a TR of 1500 ms (TE 35 ms, flip angle 90°).

### 4. fMRI Analysis

Image processing and statistical analyses were carried out using Statistical Parametric Mapping (SPM5, Wellcome Trust Centre for Neuroimaging, London, UK) with a random effects model for group analyses. Preprocessing of the individual functional scans included realignment to correct for motion artifacts, slice timing, spatial normalization to a standard template (Montreal Neurological Institute, MNI) and smoothing with an 8 mm full width at half maximum (FWHM) Gaussian kernel. Intrinsic autocorrelations were accounted for by AR(1) model and low frequency drifts were removed via high pass filtering with a cut-off frequency of 128 seconds.

After preprocessing, individual first level analyses were performed for each subject. According to the general linear model we defined regressors to analyze each of the two types of video stimuli (erotic, non-erotic). Picture stimuli were weighted with each subject’s individual rating for each picture on each scanning occasion with high values indexing highly arousing erotic pictures and low values indexing subjectively low arousing non-erotic images. One regressor comprising the parametric modulation according to these individual ratings was defined. According to their actual durations, video blocks and picture trials as well as expectation arrows for erotic and non-erotic pictures were modeled as timely extended events (20 sec for the video trials, 3–5 sec for expectation and 4 sec for picture presentation) and convolved with the hemodynamic response function. The 6 realignment parameters modeling residual motion were also included in the individual models.

For each participant contrasts of erotic minus non-erotic stimulation were calculated per each experimental condition (video clips, erotic pictures, expectation) and then propagated to second level group analyses. Within-group comparisons between activation data obtained during the mid-follicular and mid-luteal phase were conducted using separate paired t-tests on contrast images of each experimental condition. Between-group comparisons of the C and nC group at the two different hormonal states (mid-follicular, mid-luteal) were computed using separate two-samples t-tests. To ensure that contrasts between cycle phases and/or groups did not depend on stimulation irrelevant differences, these comparisons were always inclusively masked with significant (p<0.05) differences obtained from the contrast erotic minus non-erotic for the minuend (e.g. nC-L[erotic minus non-erotic] minus nC-F[erotic minus non-erotic] was inclusively masked by nC-L[erotic minus non-erotic]). Statistical maps were thresholded at p≤0.001 uncorrected for multiple comparisons. Only clusters consisting of at least 10 contiguously significant voxels are reported. Clusters of significant voxels were classified to specific brain regions using printed anatomic brain atlases [29], [30] and the software implementation of the Wakeforest University (wfu pickatlas, [31]).

To visualize the overlap of different contrasts, a conjunction analysis as implemented in SPM 5 (conjunction null [32]) was used to test for a significant effect of erotic stimulation across all the three different hormonal levels.

## Results

In subjects taking contraceptives, average estradiol levels were below assessment thresholds of 5 ng/L. In subjects without hormonal contraception estradiol levels in the follicular phase were significantly lower than in the luteal phase. Similarly, progesterone levels in the follicular phase were significantly lower than in the luteal phase and lowest in the C group (see Table 1). Subjective sexual functioning as measured by the MGH-SFQ did not differ between females of the C group and females of the nC group when sexual functioning was assessed at the two different time-points of their cycle (Table 1).

**Table 1:** Sample characteristics: Hormonal levels and questionnaires.

	nC-L	nC-F	C	nC-L vs. nC-F*p*	nC-L vs. C*p*	nC-F vs. C*p*
Estrogen (ng/L)	182.8 (49.9)	39.1 (12.7)	<5	<0.001	–	–
Progesterone (µg/L)	13.1 (7.2)	0.52 (0.29)	0.29 (0.16)	<0.001	<0.001	0.03
MGH-SFQ	2.4 (0.82)	2.5 (0.45)	2.7 (0.89)	0.72	0.52	0.67
Erotic picture ratings	6.1 (1.2)	6.2 (1.5)	5.1 (2.0)	0.67	0.15	0.14
Non-erotic picture ratings	1.4 (0.33)	1.3 (0.32)	1.2 (0.22)	0.50	0.24	0.46
ADS	6.5 (2.2)		5.9 (3.9)	–	0.63	

Values are means and standard deviations (in rounded brackets) of serum estrogen and progesterone levels, subjective ratings of sexual dysfunction as assessed by the Massachusetts General Hospital Sexual Functioning Questionnaire (MGH-SFQ), subjective ratings of erotic and non-erotic pictures as presented during fMRI (visual analogue scale 1–9 with 1: not sexually arousing at all, 9: very sexually arousing) and symptoms of depression as measured with the ADS scale in the group taking contraceptives (C) and in the group without contraceptives during the follicular (nC-F) or luteal (nC-L) phase of the menstrual cycle; *p*: significance of group differences computed from paired and unpaired t-tests.

For the picture study, average ratings of sexual arousal induced by each stimulus, did not differ between the C group and the nC group assessed mid-follicularly and mid-lutealy, neither for erotic nor non-erotic pictures (Table 1). Results from the ADS scale (see Table 1) confirmed that none of the subjects suffered from depression at the time of the study.

### 1. fMRI Results: Main Effects

**Figure 1:**
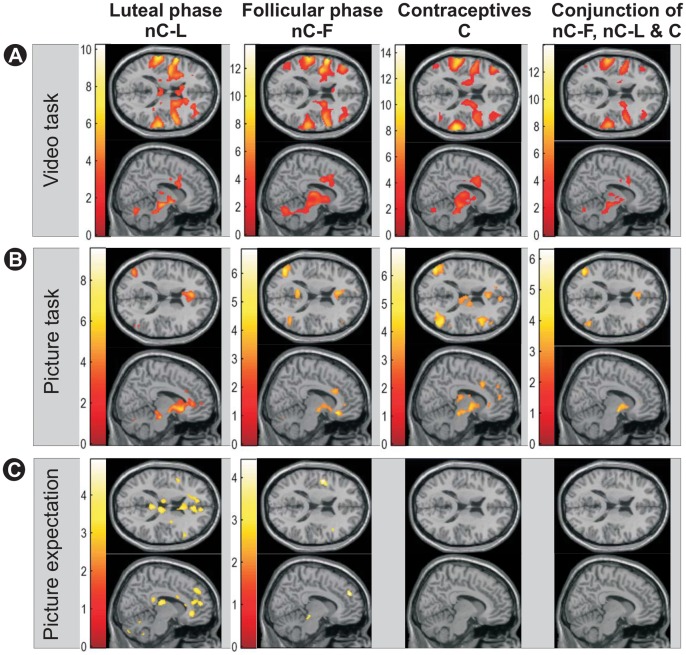
Differential brain activation contrasting erotic minus non-erotic stimulation. Depicted are contrasts from experimental runs with video (A), picture (B) and picture expectation (C) stimuli during luteal and follicular phase of the menstrual cycle and under oral contraceptives. The rightmost panel depicts the conjunction of the three conditions. For demonstration purposes, all thresholds were set at p<0.005 uncorrected. Even at such a more lenient threshold, no differential brain activation was found under contraceptives upon picture expectation.

First, we tested on the main effect of erotic stimulation elicited either by videos or pictures under the influence of hormonal oral contraceptives and the two different phases of the menstrual cycle. In all three hormonal conditions (C, nC-F, nC-L) and for both, the erotic video stimulation and picture presentation significant (p<0.05, FDR corrected) activations were observed in the precentral gyrus, insula, cingulate cortex, lateral inferior prefrontal cortex, hypothalamus, thalamus, ventral striatum, brain stem, inferior parietal lobule, and the fusiform gyrus (see Tables S1 and S2 for exact locations and significances). Consistent activation of the amygdala upon video and picture presentation was evident only under oral contraception and appeared upon picture presentation in the nC-F group. Activation of the medial prefrontal cortex was not found upon video stimulation but under all three hormonal conditions upon picture presentation. For the nC-F and nC-L groups but not the C group the medial prefrontal cortex was also activated by expectation of erotic pictures. Furthermore, picture expectation (see Table S3) activated the precentral gyrus, anterior insula and the thalamus only in the nC-F group while activations along the cingulate cortex were significant only in the nC-L group. In the C group, expectation of erotic stimuli did not yield any significant activation even at lowered thresholds down to a p-valued of p<0.005 (uncorrected). Main effects of all three experimental conditions for the different hormonal states are summarized in Figure 1A. Conjunction analyses of significant effects upon video presentation confirmed the visual impression of a strong anatomical overlap of activated regions across the three hormonal states (Figure 1A, rightmost column). For static erotic pictures rather the same brain regions were significantly activated as for the video condition, although at a considerably smaller spatial extent. Again, a conjunction analysis of significant effects confirmed a strong anatomical overlap of activated regions across hormonal states (Figure 1B, rightmost column).

### 2. fMRI Results: Group Comparisons

T-tests on between-group comparisons (C vs nC-F; C vs nC-L) as well as on the within-group comparisons (nC-F vs nC-L) separately for each stimulation type demonstrated only rather small and circumscribed interaction effects (for a summary, see Table 2).

**Table 2:** Significant condition-by-hormonal levels interaction effects (whole brain analysis).

	Comparisons within nC group			Comparisons between nC and C groups											
	nC-L>nC-F			nC-F>C			nC-L>C			C>nC-F			C>nC-L		
erotic minus non-erotic	t	NV	Peak atx/y/z	t	NV	Peak atx/y/z	t	NV	Peak atx/y/z	t	NV	Peak atx/y/z	t	NV	Peak atx/y/z
**Video:**															
Precentral gyrus BA 6/44, r				4.30	40	62/6/18*									
Precentral gyrus BA 6/44, l				3.53	9	−58/8/16*									
DLPFC BA 9/46										4.52	34	48/44/6*			
**Pictures:**															
Sup. temporal gyrus BA 22													3.35	27	60/−48/6*
**Expectation of pictures:**															
Precentral gyrus, BA 6/44, r				5.15	56	**46/14/12**									
Precentral gyrus, BA 6/44, l				6.39	214	**−46/−2/14∼**									
Insula, l				5.98	#	**−40/6/12**									
Insula, r				4.34	13	**36/4/16**									
Pregenual anterior cingulate	3.67	14	14/38/−2*												
Anterior middle cingulate	4.46	67	−8/32/32*				4.23	36	12/46/22*						
							5.29	324	**−16/40/16∼**						
Posterior middle cingulate							5.01	#	**−14/18/32**						
							3.71	25	6/8/28*						
DMPFC BA 9	4.26	23	16/44/38*	4.81	42	**10/48/30**	4.35	100	16/46/36*						
DLPFC BA 10	4.18	21	30/58/0*				4.42	78	32/42/22*						
Inferior frontal gyrus, BA 11	3.98	16	−20/40/−8*				4.11	33	−20/38/−8*						
Parahippocampus	4.51	10	24/−20/−22*												
Posterior cingulate				4.17	19	**−8/−6/34**									
Inferior parietal lobe				3.92	11	**−56/−38/30**									

nC-group: women not taking hormonal contraceptives; F: mid-follicular phase of menstrual cycle; L: mid-luteal phase of menstrual cycle; C-group: women taking hormonal contraceptives; r: right; l: left; no significant interactions for nC-F>nC-L; #: activation maximum is part of cluster above. Group comparisons surviving an FDR corrected threshold of p<0.05 are marked by MNI-coordinates in bold type (a tilde behind the z-coordinate denotes significance at p<0.05, FWE corrected); an asterisk (*) denotes group differences at an uncorrected level of p<0.001 with cluster sizes of at least 10 contiguously significant voxels; t: t-value; NV: number of contiguously significant voxels; peak coordinates of clusters are MNI (Montreal Neurological Institute) normalized stereotactic coordinates: -x: left from the anterior commissure (AC); -y: posterior from AC; -z: inferior from AC. DLPFC: dorsolateral prefrontal cortex; DMPFC: dorsal medial prefrontal cortex; BA: Brodman area.

#### 2.1. Dynamic video stimulation

For dynamic video stimulation no differences emerged when comparing the nC subjects at their different hormonal states. Group comparisons with the C group showed one cluster in the inferior part of the bilateral pre-central gyrus, however only when significance (p<0.001) was not corrected for multiple comparisons. We nevertheless report it here due to its anatomical parallels with interaction effects upon picture expectation; see below. The C group’s activation of the precentral gyrus was lower when compared against activation of the nC group during the mid-follicular phase, but not when compared against the mid-luteal phase. Conversely, the C group showed increased activation in the dorso-lateral prefrontal cortex when compared against the nC group at its mid-follicular phase, but not when compared against the mid-luteal phase.

#### 2.2. Static erotic pictures

For static erotic pictures again no within-group differences emerged (nC-F vs. nC-L), and between-group comparisons only demonstrated increased activation at uncorrected thresholds of the superior temporal gyrus under oral contraceptives when compared against the nC group at its mid-luteal but not at its mid-follicular phase.

#### 2.3. Expectation of erotic pictures

For this experimental condition main effect analyses of the expectation of erotic vs. non-erotic pictures for each hormonal level already indicated differences between both groups of C and nC subjects which appeared to be further modulated by the hormonal status of the nC group. A conjunction analysis could show that no brain region was conjointly activated across all three hormonal states under investigation (see Figure 1C, rightmost column). This absence of a conjoint effect was mainly due to the absence of a main effect of expecting erotic stimuli in the C group (see above 1.1. Main effects).

**Figure 2:**
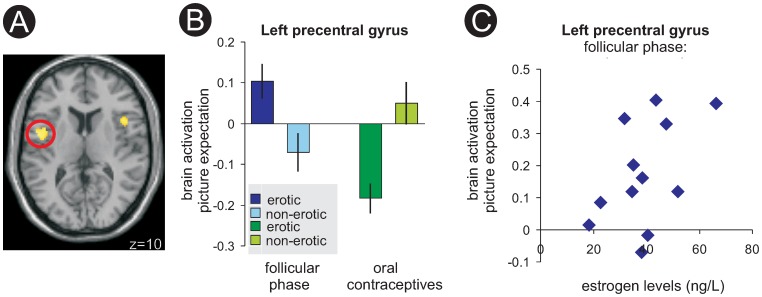
Differential brain activation in the precentral gyrus upon expectation of picture stimuli. A – differential brain activation in bilateral precentral gyrus during expectation of picture stimuli (nC-F [erotic minus non-erotic]>C [erotic minus non-erotic]) thresholded at p = 0.001 uncorrected, minimum 10 voxels per cluster. B – parameter estimates of modelled effects of brain activation in the left precentral gyrus in the follicular phase (nC-F group) and under oral contraceptives (C group) upon picture expectation. C – Illustration of the relationship between differential brain activation upon picture expectation (erotic minus non-erotic) in the precentral gyrus and estrogen levels in the nC-F group. Color coded bars represent T-levels.

Results of the between- and within group comparisons on hemodynamic responses during the expectation of erotic pictures are summarized in Table 2. These differences were most pronounced when comparing the nC group during its two different cycle-phases against the C group. Comparing the nC group during its follicular phase against the C group revealed significantly (p<0.05, corrected) higher activation for the nC-F group in left and right anterior insula, dorsal medial prefrontal cortex, and left inferior parietal lobe. This contrast also yielded a significant difference in the bilateral inferior precentral gyrus as already observed during dynamic video stimulation (Figure 2). During the luteal phase the nC showed significantly greater activation (p<0.05, corrected) upon expectation than the C group in the anterior and posterior middle cingulate cortex. Comparable to the follicular phase group differences with the C group were again evident in dorsal medial prefrontal cortex, however only at lowered significance (p<0.001, uncorrected).

Comparing both hormonal levels against each other, only the luteal phase was associated with higher activation in the anterior cingulate cortex, the dorsolateral and dorso-medial prefrontal cortex at uncorrected thresholds (p<0.001). The inverted contrast (nC-F minus nC-L) did not produce any significant phase-differences.

### 3. Correlations

To further investigate relationships of hormonal status and brain activation, individual estimates of hemodynamic responses in the left and right inferior precentral gyrus during video stimulation and picture expectation were further investigated on their extent of correlation with individual estrogen levels during the follicular phase. The average beta value of all voxels within the respective cluster as reported in table 2 was used. Since activation during picture expectation in the left precentral gyrus overlapped with insular activation, the average beta value of all voxels within a sphere of 6 mm around the maximum was used. We found a significant (r = 0.49, p = 0.05) correlation of follicular phase oestrogen and brain activation in the follicular phase during picture expectation in the left precentral gyrus (Figure 2). Left precentral activation in the follicular phase during the video task also showed a positive correlation with estrogen levels (r = 0.41, p = 0.09) that however failed significance.

## Discussion

In the present study we investigated the neural correlates of erotic processing in healthy female volunteers taking oral contraceptives as compared to women in early follicular or mid-luteal phase (not taking contraceptives). Direct erotic stimulation with either dynamic videos or static pictures induced significant brain activation in a set of brain regions which has already been described by previous studies using comparable erotic stimuli [10], [11], [13], [14], [33], [34], [35], [36]. Interestingly, this rather direct confrontation with explicit visual erotic stimuli at given thresholds was only associated with few differences in quite distinct brain regions between the different hormonal states and under oral contraceptives. Only in the bilateral inferior part of the precentral gyrus the nC group in its follicular phase demonstrated higher activation when compared against the C group.

A different pattern emerged for less direct erotic stimulation, experimentally induced by investigating brain activation upon expectation of static erotic stimuli. This experimental condition was associated with considerably less anatomical overlap of activated brain regions across the different hormonal states. Instead, activation differences already emerged when comparing the nC group at its different phases. Group differences were most pronounced when comparing the nC group during both hormonal states against the C group. Among the various brain regions showing either within- or between-group differences, the dorso-medial prefrontal cortex consistently emerged in three different contrasts comparing the luteal against the follicular phase in the nC group, and also when comparing both phases against the C group. Across tasks (video stimulation, expectation of erotic pictures), the inferior pre-central gyrus was consistently more activated in the nC group during its follicular phase than in the C group.

Women’s interest in visual sexual stimuli has been shown to be modulated by hormonal states [37], [38]. In a study using eye-tracking methods to measure eye movements to different aspects of erotic pictures, it was observed that women who did not take oral contraceptives spent more time looking at genitals whereas women with oral contraceptives focused more on contextual details such as clothing or background [39].

The rationale for the present study was to further investigate these effects of different hormonal levels corresponding to the mid-follicular and mid-luteal phase of the menstrual cycle and during oral contraception. A better understanding of possible hormonal moderators of the neural correlates of erotic stimulation in women may help to understand other influences such as processing of erotic stimuli under different anti-depressants. From the present results it appears that different hormonal levels do exert only minor effects on these neural correlates when visual erotic stimulation is rather direct and explicit. Particularly, subcortical regions that were most affected by antidepressant treatment in males [19] did not show marked differences associated with different hormonal levels. We therefore conclude that in comparison to psychotropic drugs that are known to affect sexual arousal such as SSRI antidepressant drugs, oral contraceptives have much weaker effects, and women taking contraceptives may be well suited for pharmaco-fMRI trials as long as erotic stimulation is direct and explict. Also the absence of differences in subjectively rated sexual functioning adds to this conclusion. It is of note, however, that from the effects related to the two phases of the menstrual cycle investigated in the present study, no conclusions can be drawn regarding effects around ovulation. Because of the inconsistent results concerning brain activation around the ovulatory phase [14], [16], we decided to assess female brain activation related to sexual stimulation at two time points clearly apart from ovulation and compare these to the activation under oral contraceptives.

While brain activation upon direct visual erotic stimulation was not markedly affected by different hormonal levels this appears not to be the case when expectation of erotic stimuli was induced as a separate condition. For this condition significant hormonal influences on activated brain regions were observed. However, it remains unclear whether these differences indicate a state of reduced sexual appetence under hormonal contraceptives since results from previous studies also have demonstrated effects of contraceptives on brain activation during a pure cognitive task (verb generation task; [40]). Interestingly though, highly significant cortical group differences between the C group and the nC group were most pronounced when comparing effects of contraceptives with those in the mid-follicular phase on expectation of erotic pictures. Since increasing brain activation upon erotic stimulation has been related to rising estrogen levels during the follicular phase [14] and also under hormonal replacement [15], these differences may still relate to reduced sexual appetence under oral contraceptives.

This interpretation is further supported by the bilaterally reduced activation of the pre-central gyrus under oral contraception, that was evident during both the dynamic video stimulation and the picture expectation task when compared against the nC group mid-follicularly. The precentral gyrus is a brain area with a high density of estrogen receptors [41] and has greater relative volume in women than in men [42]. In men, activation of the pre-central gyrus has already been related to measures of penile turgidity under erotic vs. non-erotic visual stimulation [43]. In a study on the relationship of women’s preference for masculine traits and phases of the menstrual cycle, greater responsiveness of the pre-central gyrus was found for masculine faces during the follicular phase [44]. Together with the posterior cingulate cortex, pre-central gyrus activation has also been associated with scores of sexual inhibition, and the authors of that study identified the pre-central gyrus as one of the brain regions associated with behavioural inhibition and self-awareness in wider contexts [44]. Furthermore, the posterior cingulate cortex but also the pre-central gyrus have been shown to be involved in mental simulation of actions [45] and self-recognition [46]. In addition to our present observation these results suggest that the pre-central gyrus may have something to do with the self-related anticipation of a sexual action. Its decreased activation under hormonal contraceptives may therefore indicate a decreased motivation or drive towards sexual activity under these drugs. Similar interpretations may apply for the adjacent differential insular activations that partially overlapped with precentral clusters for the same contrast. Particularly the left-sided insula has been related to mental representations of subjective pleasure upon sexual experiences in women [47].

Although as a statistical trend (contrasts survived uncorrected but not corrected thresholds), the oral contraceptive group’s activation upon expectation of erotic stimuli was reduced compared to mid-luteal nC subjects in two additional brain regions, the dorsomedial prefrontal cortex (DMPFC) and anterior middle cingulate cortex. Using the same picture stimuli as in the present study, DMPFC activation upon erotic stimulation has been repeatedly reported in previous studies [36], [48]. Although particularly anterior parts of the DMPFC could be linked to the processing of erotic stimuli, those previous data favoured a more general role of this region in the context of attending to mental states or processing of socially relevant stimuli, particularly those with high self-relevance [49], [50]. By contrast, involvement of the anterior middle cingulate cortex appears to rely more specifically on sexual stimulation, since activation of this region was correlated with penile tumescence as shown by recent studies using positron-emission tomography [35], fMRI [51], and arterial spin labelling measures [52].

Regarding limitations to our study, the actual sample size of 12 subjects per group may confine generalizability of the results. Also, we did not obtain objective measures of sexual arousal and functioning. Thus we could not objectively infer effects of contraceptives on sexual functioning in women, beyond subjective rating data. Therefore, while our experimental data may allow for inferences regarding hormonal effects on brain responsivity upon erotic stimulation, the relation to actual sexual functioning should be subject to further investigations. Furthermore, the video material used was most likely produced for a male audience and was implemented for reasons of comparability with the results of our previous studies. Currently, we cannot answer the question of how brain activation in female brains would compare to present results if the video material had been tailored specifically for a female audience.

Taken together, different hormonal states along the menstrual cycle or the intake of oral contraceptives predominantly modulated activation of cortical brain regions upon expectation of erotic stimuli. During direct and explicit erotic stimulation, either by dynamic videos or static pictures the effects of hormonal influences appeared to be comparably weak. Most likely, the strong overall effect of this kind of erotic stimulation may suppress subtle effects of different hormonal states during menstrual cycle or under oral contraceptives and hormonal effects on brain activation during direct and explicit erotic stimulation may not interfere with stronger influences such as medical conditions or psychotropic drugs. We therefore conclude that each of the hormonal states investigated can be selected to study psychopharmacological effects on brain activation during erotic stimulation as long as all women under investigation adhere to the same hormonal state. It is of note, however, that it should not be concluded from present data that different hormonal states can be pooled within one sample.

## Supporting Information

Figure S1 **Erotic video clip task.** Subjects were instructed to passively watch erotic and non-erotic video clips from commercial adult videos presented in a randomized order for 20 sec each.
(PDF)

Figure S2 **Erotic picture task.** Subjects were instructed to passively watch 20 erotic and 20 non-erotic pictures from the International Affective Picture System for 4 sec each whereas half of each type of pictures was announced by arrows for 3–5 sec.
(PDF)

Table S1 **Main effects (whole brain analysis): Videos.**
(PDF)

Table S2 **Main effects (whole brain analysis): Pictures.**
(PDF)

Table S3 **Main effects (whole brain analysis): Expectation of pictures.**
(PDF)
