# Supplementary figures and images for: Neural Correlates of Erotic Stimulation under Different Levels of Female Sexual Hormones
Source: PLoS One. 2013 Feb 13;8(2):e54447. doi: 10.1371/journal.pone.0054447 (PMC3572100; doi:10.1371/journal.pone.0054447)

**Supplementary Figure S1:** Erotic video clip task

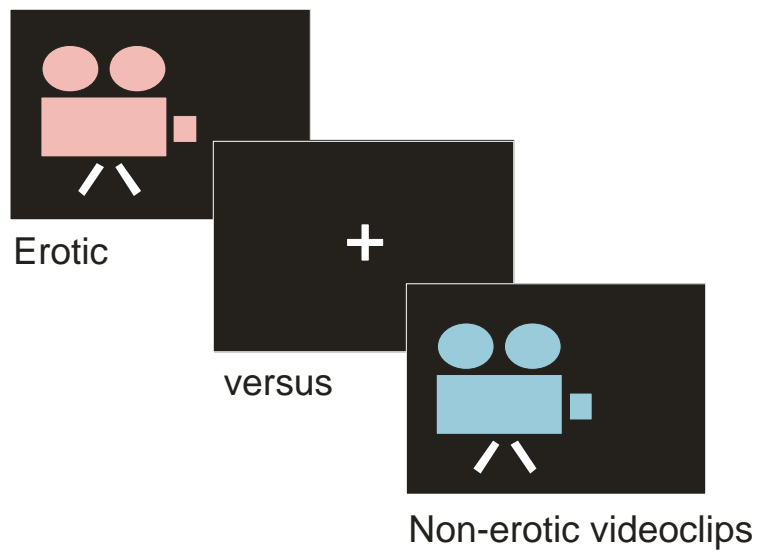

Supplement: Figure S1 — Erotic video clip task. Subjects were instructed to passively watch erotic and non-erotic video clips from commercial adult videos presented in a randomized order for 20 sec each. (PDF) [file pone.0054447.s001.pdf]
