# Supplementary material for: Neural Correlates of Erotic Stimulation under Different Levels of Female Sexual Hormones
Source: PLoS One. 2013 Feb 13;8(2):e54447. doi: 10.1371/journal.pone.0054447 (PMC3572100; doi:10.1371/journal.pone.0054447)

Supplementary Figure S2: Erotic picture task

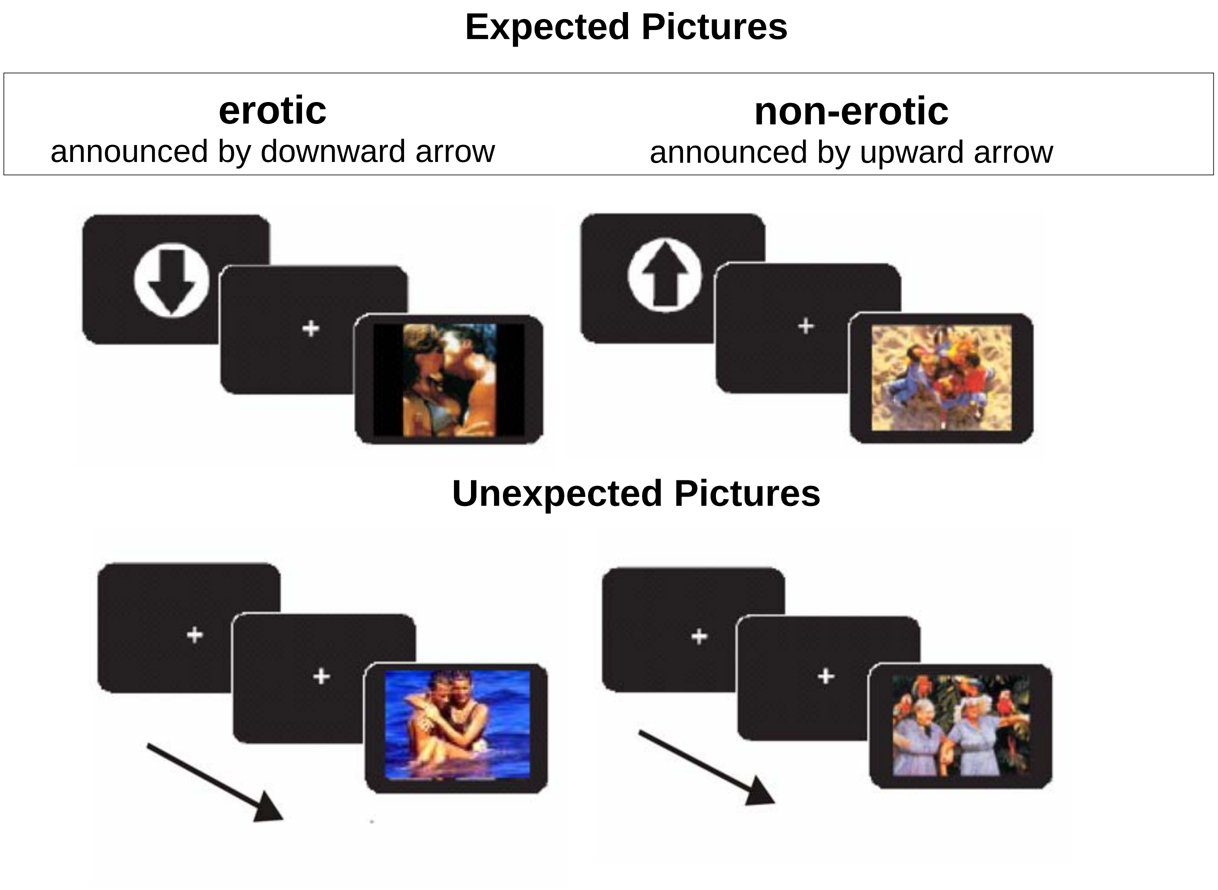

Supplement: Figure S2 — Erotic picture task. Subjects were instructed to passively watch 20 erotic and 20 non-erotic pictures from the International Affective Picture System for 4 sec each whereas half of each type of pictures was announced by arrows for 3–5 sec. (PDF) [file pone.0054447.s002.pdf]
