# Supplementary material for: Neural Correlates of Erotic Stimulation under Different Levels of Female Sexual Hormones
Source: PLoS One. 2013 Feb 13;8(2):e54447. doi: 10.1371/journal.pone.0054447 (PMC3572100; doi:10.1371/journal.pone.0054447)
Supplement: Table S1 — Main effects (whole brain analysis): Videos. (PDF) [file pone.0054447.s003.pdf]

Table S1: Main effects (whole brain analysis): Videos

erotic minus non-erotic	nC-F group t	nC-F group p	nC-F group Peak at x/y/z	nC-L group t	nC-L group p	nC-L group Peak at x/y/z	C group t	C group p	C group Peak at x/y/z
**Cortical structures**									
Precentral gyrus, BA 6/44, l	10.44	<0.001	-56/6/16	7.37	<0.001	-56/6/18~	6.74	<0.001	-54/6/22
Precentral gyrus, BA 6/44, r	7.30	<0.001	52/4/12	5.40	<0.001	48/2/14	6.92	<0.001	48/6/24
	7.72	<0.001	56/8/32	6.36	<0.001	54/8/26~	8.59	<0.001	54/4/34
Insula, l	9.40	<0.001	-40/-2/10	6.78	<0.001	-36/-4/12~	9.81	<0.001	-38/-12/-6
Insula, r	8.15	<0.001	42/8/-8	7.00	<0.001	40/6/-10~	9.86	<0.001	38/-4/-12
Anterior insula/inferior frontal gyrus, r	6.96	<0.001	30/20/8	5.22	<0.001	38/22/8	6.97	<0.001	36/30/6
Anterior insula/inferior frontal gyrus, l	7.36	<0.001	-34/22/8	5.75	<0.001	-30/16/12~	6.88	<0.001	-36/22/4
Posterior middle cingulate	7.01	<0.001	2/14/28	4.21	<0.001	-4/18/30	7.42	<0.001	2/10/28
Posterior cingulate	7.64	<0.001	6/-2/34	5.69	<0.001	2/4/32~	7.00	<0.001	-4/-4/36
DLPFC BA 9/46, l	6.49	<0.001	-46/38/18	4.18	<0.001	-36/40/28	7.07	<0.001	-44/36/12
DLPFC BA 9/46, r	6.22	<0.001	50/38/14	3.38	<0.001	42/44/18	9.39	<0.001	50/42/6
Inferior parietal lobe, r	11.57	<0.001	60/-32/20	7.50	<0.001	62/-30/22~	14.03	<0.001	58/-32/20
Inferior parietal lobe, l	13.63	<0.001	-60/-34/28	10.91	<0.001	-60/-36/-28~	14.50	<0.001	-60/-34/28
Fusiform gyrus/inferior temporal gyrus, BA 19/37, r	8.44	<0.001	46/-58/-12	7.00	<0.001	46/-58/-10~	9.31	<0.001	46/-58/-12
Fusiform gyrus/inferior temporal gyrus, BA 19/37, l	8.78	<0.001	-40/-68/-8	7.19	<0.001	-44/-70/-8~	8.76	<0.001	-40/-68/-8
**Subcortical structures**									
Thalamus, dorsomedian Nc, l	6.23	<0.001	-6/-12/2				4.81	<0.001	-4/-12/0
Thalamus, dorsomedian Nc, r	6.01	<0.001	8/-18/8	4.34	<0.001	0/-14/8	5.46	<0.001	8/-18/8
Thalamus, ventrolateral Nc, r							7.81	<0.001	18/-12/12
Nucleus accumbens, r	4.92	<0.001	14/4/-2						
Nucleus accumbens, l	4.37	<0.001	-8/4/-4						
Brainstem/Tegmental area	6.67	<0.001	-6/-28/-14	5.73	<0.001	8/-22/-14~	6.30	<0.001	-8/-20/-12
Hypothalamus, r				5.49	<0.001	12/-4/-8			
Hypothalamus, l							4.92	<0.001	-6/-4/-6
Substantia nigra/ subthalamic nucleus, l	6.59	<0.001	-14/-10/-8	6.09	<0.001	-16/-8/-8~	4.28	<0.001	-14/-8/-8
Substantia nigra/ subthalamic nucleus, r	5.91	<0.001	14/-10/-8						
Amygdala, r							6.21	<0.001	22/-2/-14
Amygdala, l							6.23	<0.001	-22/-4/-16
**Cerebellum**									
	9.19	<0.001	-26/-70/-26	5.81	<0.001	-28/-70/-26~	7.45	<0.001	-30/-60/-26
	7.73	<0.001	-28/-62/-28						
	7.67	<0.001	24/-66/-28	5.83	<0.001	26/-66/-28~	4.87	<0.001	24/-66/-26

All activations in Supplemental Table 1 survived FDR corrections at p<0.05; all activations in the nC-F and C group and those marked with "~" in the nC-L group survived FWE corrections at p<0.05.
nC-group: women not taking hormonal contraceptives; F: mid-follicular phase of hormonal cycle; L: mid-luteal phase of menstrual cycle; C-group: women taking hormonal contraceptives; r: right; l: left
No significant interactions for nC-F > nC-L
T: t-value; NV: number of contiguously significant voxels; peak coordinates of clusters are MNI (Montreal Neurological Institute) normalized stereotactic coordinates: -x: left from the anterior commissure (AC); -y: posterior from AC; -z: inferior from AC. DLPFC: dorsolateral prefrontal cortex; (D)MPFC: (dorsal) medial prefrontal cortex; BA: Brodman area; Nc: Nucleus
Activations with a minimum number of voxels of at least 10 are reported down to a significance threshold of p<0.005. We chose this more lenient threshold because we wanted to ascertain that functional activations in any of the groups were not masked out by a too conservative thresholding.
