# Supplementary material for: Neural Correlates of Erotic Stimulation under Different Levels of Female Sexual Hormones
Source: PLoS One. 2013 Feb 13;8(2):e54447. doi: 10.1371/journal.pone.0054447 (PMC3572100; doi:10.1371/journal.pone.0054447)
Supplement: Table S2 — Main effects (whole brain analysis): Pictures. (PDF) [file pone.0054447.s004.pdf]

Table S2: Main effects (whole brain analysis): Pictures

	nC-F group			nC-L group			C group		
**erotic minus non-erotic**	**t**	**p**	**Peak at x/y/z**	**t**	**p**	**Peak at x/y/z**	**t**	**p**	**Peak at x/y/z**
**<u>Cortical structures</u>**									
Precentral gyrus, BA 6/44, l	4.13	<0.001	-46/0/28	3.56	=0.001	-54/6/26	3.47	=0.001	-46/0/28
Precentral gyrus, BA 6/44, r	4.09	<0.001	42/8/22	4.17	<0.001	50/12/28	5.38	<0.001	44/6/28
							5.51	<0.001	48/16/24
Insula, r	3.44	=0.001	42/12/-8						
Anterior insula/inferior frontal gyrus, r	5.38	<0.001	40/26/-16	4.53	<0.001	38/30/8	4.54	<0.001	34/18/-8
	3.20	=0.002	36/32/4						
Anterior insula/inferior frontal gyrus, l	3.39	=0.001	-24/32/-12				4.77	<0.001	-32/20/8
	4.07	<0.001	-30/24/12						
Subgenual cingulate	5.95	<0.001	4/30/-12~	4.16	<0.001	4/34/-10	4.58	<0.001	2/30/-10
Pregenual cingulate	4.92	<0.001	-6/36/4	5.88	<0.001	-2/38/12	3.94	<0.001	2/32/6
Anterior middle cingulate	4.16	<0.001	-6/24/22	4.07	<0.001	-4/26/24	3.84	<0.001	0/28/16
Posterior middle cingulate	3.98	<0.001	0/12/38				4.64	<0.001	2/14/34
Posterior cingulate	4.58	<0.001	-10/-46/18				4.10	<0.001	2/-10/34
	3.45	=0.001	4/-18/34						
	3.27	=0.002	2/-2/32						
DMPFC, BA 9	3.31	=0.002	4/50/38				3.96	<0.001	16/48/34
MPFC, BA 9	3.90	<0.001	-2/54/-2	4.52	<0.001	0/56/0	3.78	=0.001	6/50/12
DLPFC BA 9/46, r							5.33	<0.001	52/40/2
Inferior parietal lobe, l	5.60	<0.001	-46/-70/18						
Postcentral gyrus, l	4.86	<0.001	-56/-22/34	9.51	<0.001	-56/-24/34~	5.06	<0.001	-54/-24/34
Postcentral gyrus, r				4.88	<0.001	60/-20/34	4.44	<0.001	58/-22/34
Fusiform gyrus inferior temporal gyrus, BA 19/37, r	6.64	<0.001	48/-70/-12~	7.31	<0.001	44/-76/0~	6.93	<0.001	38/-66/-6~
Fusiform gyrus inferior temporal gyrus, BA 19/37, l	6.40	<0.001	-48/-64/2~	7.71	<0.001	-52/-68/-6~	6.44	<0.001	-50/-64/2~
**<u>Subcortical structures</u>**									
Thalamus, dorsomedian Nc, l	3.24	=0.002	-2/-6/8	3.50	<0.001	-2/-14/4			
Thalamus, dorsomedian Nc, r	3.42	=0.001	4/-16/12				4.06	<0.001	4/-16/14
Thalamus, ventrolateral Nc, r									
Nucleus accumbens, r	3.28	=0.002	8/14/-6	5.82	<0.001	8/16/-4~	4.51	<0.001	4/2/-2
Nucleus accumbens, l	2.84	=0.004	-6/8/-6	5.11	<0.001	-8/16/-4	3.76	<0.001	-10/6/-6
Brainstem/Tegmental area	3.64	=0.001	6/-26/-18	6.61	<0.001	8/-24/-20	3.84	<0.001	8/-20/-10
Hypothalamus, r	4.61	<0.001	6/-8/-6	6.19	<0.001	6/-6/-6	4.28	<0.001	8/-4/-4
Hypothalamus, l	4.50	<0.001	-8/-8/-12	6.11	<0.001	-8/-8/-12	4.56	<0.001	-6/-6/-2
							4.34	<0.001	-8/-8/-12
Substantia nigra/ subthalamic nucleus, l				4.06	<0.001	-12/-18/-12			
Substantia nigra/ subthalamic nucleus, r	5.46	<0.001	16/-16/-12	3.78	<0.001	16/-18/-12	3.82	<0.001	14/-16/-12
Amygdala, r							3.12	=0.001	22/-6/-14
Amygdala, l	3.73	=0.001	-24/-4/-18				3.28	=0.002	-24/-6/-16
**<u>Cerebellum</u>**									
	6.81	<0.001	-42/-56/-22	4.62	<0.001	-26/-62/-34	5.83	<0.001	-12/-76/-36
	4.22	<0.001	-34/-78/-38	4.74	<0.001	46/-54/-34			
	3.83	<0.001	-10/-76/-38	3.70	=0.001	20/-70/-26			

All activations in Supplemental Table 2 survived FDR corrections at p<0.05, activations marked with "~"survived also FWE corrections at p<0.05.
nC-group: women not taking hormonal contraceptives; F: mid-follicular phase of hormonal cycle;
L: mid-luteal phase of menstrual cycle; C-group: women taking hormonal contraceptives; r: right; l: left
No significant interactions for nC-F > nC-L
T: t-value; NV: number of contiguously significant voxels; peak coordinates of clusters are MNI (Montreal Neurological Institute) normalized stereotactic coordinates: -x: left from the anterior commissure (AC); -y: posterior from AC; -z: inferior from AC. DLPFC: dorsolateral prefrontal cortex; (D)MPFC: (dorsal) medial prefrontal cortex; BA: Brodman area; Nc: Nucleus
Activations with a minimum number of voxels of at least 10 are reported down to a significance threshold of p<0.005. We chose this more lenient threshold because we wanted to ascertain that functional activations especially in the C group were not masked out by a too conservative thresholding.
