# Supplementary material for: Neural Correlates of Erotic Stimulation under Different Levels of Female Sexual Hormones
Source: PLoS One. 2013 Feb 13;8(2):e54447. doi: 10.1371/journal.pone.0054447 (PMC3572100; doi:10.1371/journal.pone.0054447)
Supplement: Table S3 — Main effects (whole brain analysis): Expectation of pictures. (PDF) [file pone.0054447.s005.pdf]

Table S3: Main effects (whole brain analysis): Expectation of pictures

	nC-F group			nC-L group			C group		
**erotic minus non-erotic**	**t**	**p**	**Peak at x/y/z**	**t**	**p**	**Peak at x/y/z**	**t**	**p**	**Peak at x/y/z**
**Cortical structures**									
Precentral gyrus, BA 6/44, r	3.24	=0.001	44/16/12						
Precentral gyrus, BA 6/44, l	4.40	<0.001	-46/-2/16						
Anterior insula/inferior frontal gyrus, l	3.54	<0.001	-28/26/-2						
Pregenual anterior cingulate				3.35	=0.001	12/34/-6			
Anterior middle cingulate				3.27	=0.001	-16/38/18			
Posterior middle cingulate				3.27	=0.001	-16/18/28			
Posterior cingulate				3.79	<0.001	4/-40/16			
DMPFC BA 9	4.25	<0.001	8/48/30	3.53	<0.001	12/44/38			
DLPFC, BA 9/46				2.98	=0.002	32/34/24			
MPFC BA 9				3.14	=0.002	10/52/14			
Inferior frontal gyrus, BA 11				3.10	=0.002	-22/42/-8			
**Subcortical structures**									
Thalamus, dorsomedian Nc	4.15	<0.001	0/-10/0						
Dorsal brainstem	3.27	=0.001	8/-26/-10						
**Cerebellum**									
	4.12	<0.001	14/-78/-3	3.42	=0.001	-16/-82/-38			
	4.12	<0.001	-18/-46/-30	3.27	=0.001	-38/-64/-36			

nC-group: women not taking hormonal contraceptives; F: mid-follicular phase of hormonal cycle; L: mid-luteal phase of menstrual cycle; C-group: women taking hormonal contraceptives; r: right; l: left

No significant interactions for nC-F > nC-L

T: t-value; NV: number of contiguously significant voxels; peak coordinates of clusters are MNI (Montreal Neurological Institute) normalized stereotactic coordinates: -x: left from the anterior commissure (AC); -y: posterior from AC; -z: inferior from AC. DLPFC: dorsolateral prefrontal cortex; (D)MPFC: (dorsal) medial prefrontal cortex; BA: Brodman area; Nc: Nucleus

Activations with a minimum number of voxels of at least 10 are reported down to a significance threshold of p<0.005. We chose this more lenient threshold because we wanted to ascertain that functional activations especially in the C group were not masked out by a too conservative thresholding.
